# New Insights into the Diurnal Rhythmicity of Gut Microbiota and Its Crosstalk with Host Circadian Rhythm

**DOI:** 10.3390/ani12131677

**Published:** 2022-06-29

**Authors:** Hongyu Wang, He Zhang, Yong Su

**Affiliations:** 1 Laboratory of Gastrointestinal Microbiology, Jiangsu Key Laboratory of Gastrointestinal Nutrition and Animal Health, College of Animal Science and Technology, Nanjing Agricultural University, Nanjing 210095, China; 2019205029@njau.edu.cn (H.W.); 2018205020@njau.edu.cn (H.Z.); 2 National Center for International Research on Animal Gut Nutrition, Nanjing Agricultural University, Nanjing 210095, China

**Keywords:** diurnal rhythmicity, gut microbiota, circadian rhythm, chronotherapy

## Abstract

**Simple Summary:**

There is a growing consensus that the gut microbiota exhibits diurnal oscillation. The rhythmicity of gut microbiota has fundamental implications for host physiology, metabolism, and health. Further, the gut microbiota rhythmicity can regulate the host’s circadian rhythm. Therefore, in this review, we aimed to highlight the rhythmic phenomenon of the gut microbiota and elucidate its fundamental roles in host physiology, metabolism, and health, and illuminate the possible interactions between the gut microbiota rhythmicity and host circadian rhythm. Insights into these questions facilitate the development of chronotherapy.

**Abstract:**

Unlike the strictly hierarchical organization in the circadian clock system, the gut microbiota rhythmicity has a more complex multilayer network of all taxonomic levels of microbial taxa and their metabolites. However, it is worth noting that the functionality of the gut microbiota rhythmicity is highly dependent on the host circadian clock and host physiological status. Here, we discussed the diurnal rhythmicity of the gut microbiota; its crucial role in host physiology, health, and metabolism; and the crosstalk between the gut microbial rhythmicity and host circadian rhythm. This knowledge lays the foundation for the development of chronotherapies targeting the gut microbiota. However, the formation mechanism, its beneficial effects on the host of gut microbial rhythmicity, and the dynamic microbial–host crosstalk are not yet clear and warrant further research.

## 1. Introduction

The rotation of the earth shapes the periodic changes of the environmental cues including light and temperature, which are tightly correlated to the physiology and metabolism of mammals. The circadian clock has thus evolved to adjust to the periodic changes of the environmental cues. The circadian clock, an intrinsic timing system with an approximate 24-h period, orchestrates the physiological functions and activities like eating, sleep-wake, and hormone secretion, and further synchronizes these activities with the changing environment [1]. The circadian timekeeper was initially supposed to only exist in higher organisms, including animals and plants. Bacteria is considered too simple to develop a circadian clock. Nevertheless, evidence illuminates that a diurnal rhythm exists in cyanobacteria involved in photosynthesis and nitrogen fixation [2,3]. Three clock proteins, including KaiA, KaiB, and KaiC, make up the circadian clock of cyanobacteria [4]. However, feedback loops of transcription are required to maintain the proper function of the oscillator [5]. More recently, the circadian oscillation was also found in the growth of *Pseudomonas putida* in the soil [6] and *Bacillus subtilis* [7], which is widely used as a probiotic additive in the animal production industry. Increasing studies reveal that the gut microbiota also exhibits robust diurnal rhythmicity at the compartmental, compositional, and functional levels in mice and humans [8,9,10,11,12]. As the primary microbial metabolites, the short-chain fatty acids (SCFAs) also undergo rhythmic oscillations [13,14,15]. However, the mechanism for the formation of microbial rhythmicity is not yet understood. Notably, the deletion of the host circadian clock gene resulted in the disrupted rhythmicity of the gut microbiota [10,16]. Whereas the disrupted gut microbiota rhythmicity was rescued by changing the feeding pattern. However, although playing a crucial role in maintaining the diurnal rhythmicity of gut microbiota, the host circadian clocks were not direct determinants of the gut microbial rhythmicity [10].

Interestingly, the gut microbiota rhythmicity could affect the normal function of the circadian clock [17]. These results suggest that the rhythmicity of gut microbiota is inextricably intertwined with the host circadian rhythm. Yet, the complex interactions between the gut microbiota rhythmicity and host biological circadian rhythm are still not fully understood. So far, the indispensable roles of the gut microbiota on the host’s metabolism, nutrition, and immunity have been repeatedly demonstrated [18,19,20,21,22]. However, the impact of gut microbiota rhythmicity on host physiology and metabolism is unclear. Therefore, the present review attempted to highlight the rhythmic phenomenon of the gut microbiota, elucidate its fundamental roles in host health, and metabolism, and illuminate the possible interactions between the gut microbiota rhythmicity and the host rhythm.

## 2. The Diurnal Rhythmicity of Gut Microbiota

In recent years, abundant evidence has indicated that the gut microbiota, its distribution, functions, and metabolites, underwent robust fluctuations [8,9,10,11,13,23]. The oral microbiome of humans also exhibited rhythmicity over a day [24]. More specifically, populations from different geographical areas have differential microbial fluctuation modes [12]. Further, similar to the compartmentalization character of microbial composition, the diurnal rhythmicity of the gut microbiota also exhibited inconsistencies between different regions of the intestine [13]. Unexpectedly, even in the same intestinal segment, the diurnal pattern of the luminal microorganisms differed from that of mucosal microbiota. Besides, the microscopic observations and quantitative PCR results have confirmed that more bacteria colonized the epithelial layer in the dark phase than in the light phase in mice [10]. It is worthy to note that different taxa dominated the gut microbial community at different times in the day. Firmicutes and Bacteroidetes were respectively prevalent at day and night in humans and mice [12,16]. As one of the most important commensal bacteria, the relative abundance of *Lactobacillus* was higher in the daytime than that in the nighttime [8]. In addition, over 20% of the microbial genes exhibited robust oscillations [8,10]. Regarding bacterial functions, pathways concerning DNA repair, cell growth, and energy metabolism were higher in the dark phase, while pathways related to detoxification, motility, and environmental sensing bloomed during the light phase [8]. Consistently, SCFAs also exhibited diurnal fluctuation, especially for acetate and butyrate [17].

## 3. The Influencers of the Gut Microbial Rhythmicity

Just like the gut microbiota configuration, the gut microbiota rhythmicity is affected by many factors such as nutritional factors (including nutrients level, antibiotics, feed additives, and diet composition), management factors (including feeding time and lighting regime), environmental factors, as well as host circadian rhythmicity and host physiology. The host circadian clock undoubtedly affects microbial rhythmicity. Related content will be discussed in the following section. Remarkably, high-fat, high-sucrose, and antibiotic-supplement diets have attenuated the rhythmic oscillation of the gut microbiota [9,10,25]. As a primary sulfur amino acid, methionine is an essential amino acid and has fundamental implications for maintaining energy-metabolism homeostasis. A methionine-restricted diet has potently alleviated inflammation in obese mice. Interestingly, the methionine-restricted diet has improved the disrupted rhythmicity of the gut microbiota induced by a high-fat diet [15]. Light is one of the most important determinants of host circadian rhythmicity. A reversing lighting regime has led to an antiphase oscillation of the most dominant microbe. Whereas a constantly dark regime has reduced the quantities of cyclical OTUs [26,27]. Further, Lu and Lee (2019) found that the light regime may entrain the rhythmicity of gut microbiota through intrinsically photosensitive retinal ganglion cells [28]. Also, supplementation of oolong tea extract can partially rescue the lost rhythmicity caused by the constantly dark regime [27]. Besides, gender is a possible factor affecting the rhythmicity of the intestinal microbiota. As one of the most dominant phyla, the diurnal rhythmicity of Bacteroidetes in relative abundance was more robust in female mice than in male mice [16]. Moreover, possible physiological and pathophysiological factors were also related to the gut microbiota rhythmicity. It is interesting to note that the rhythmicity of the oral microbiome was abolished when incubating the saliva in vitro, which implies the indispensable role of the host in maintaining the normal microbial rhythmicity [24]. In addition, individuals suffering from obstructive sleep apnea underwent an abnormal microbial oscillating pattern and metabolome [29]. Notably, compared with normal healthy people, people with obesity had damping rhythmicity of gut microbiota [12]. Sleeve gastrectomy is the most popular bariatric procedure worldwide. The dampened diurnal oscillation of gut microbiota in the obese mice induced by a high-fat diet was improved by the sleeve gastrectomy [30].

**Figure 1:**
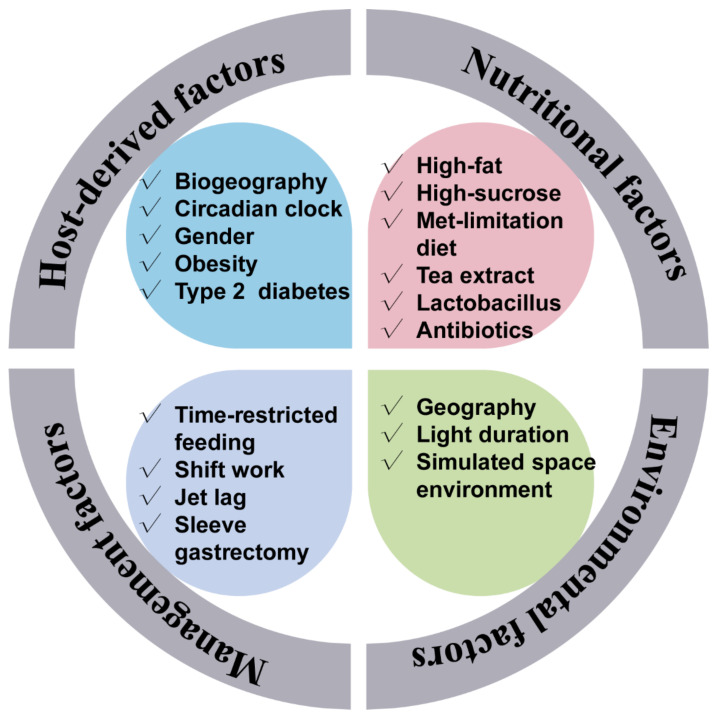
Factors that affect the gut microbiota rhythmicity. The gut microbiota rhythmicity was susceptible to many factors such as nutritional factors (including diet composition, nutrient levels, antibiotics, as well as feed additives), management factors (including feeding time, lighting regime), environmental factors, as well as host-derived factors.

Interestingly, certain environmental factors would also affect the normal gut rhythmicity. For example, in a simulated space environment, the integrated low air pressure, noise, and weightlessness condition has dissimilatory impacts on the diurnal oscillation of gut microbiota [31]. The influencers of the gut microbiota rhythmicity are summarized in Figure 1.

## 4. The Gut Microbiota Rhythmicity Is Dynamically Intertwined with the Host Circadian Rhythm

**Figure 2:**
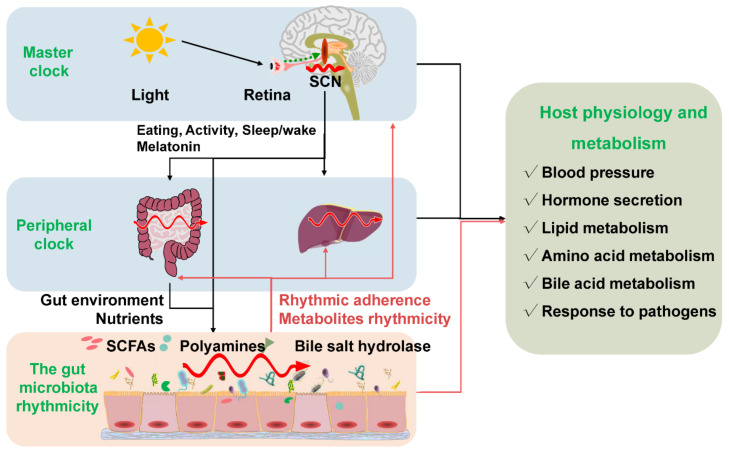
The gut microbiota rhythmicity is dynamically intertwined with the host’s circadian rhythm. The gut microbial rhythmicity works together with the host circadian system to maintain the normal rhythmicity of the host physiology and metabolism. Entrained by environmental factors (especially light cues), the master clock located in the SCN drives the host activities, such as eating behaviors, and sleep/wake rhythms to synchronize with the environment’s rhythmic changes. Through the microbiota–gut–brain axis and the gut–liver axis, the host master clock affects the rhythmicity of the gut microbiota and its metabolites (including SCFAs, polyamines, bile salt hydrolase). Interestingly, the gut microbiota and their metabolites could react to the normal transcriptomes and metabolism of the host circadian clock systems, including the master clock and the peripheral clock. SCN = suprachiasmatic nucleus; SCFAs = short-chain fatty acids.

Circadian rhythmicity is an important physiological phenomenon that plays an important role in maintaining metabolic homeostasis and sustaining normal life in prokaryotes and eukaryotes. The circadian clock in mammals consists of a master clock located in the suprachiasmatic nucleus of the hypothalamus and peripheral clocks located in other tissues of the body such as the liver and intestine [32]. The mammalian circadian clock is a self-sustaining, interlocked transcription-translation feedback loop of a network of genes including *Bmal1*, *Clock*, period circadian protein gene (*Per1*, *Per2*, and *Per3*), and cryptochrome gene (*Cry1* and *Cry2*) [33]. Intriguingly, the microbiota in the intestine can co-evolve with the host and play an indispensable role in host physiology and metabolism. Importantly, intestinal dysbiosis and circadian rhythm disruption are associated with obesity, metabolic syndrome, and inflammatory bowel disease. The light signal is the main stimulus synchronizing the master clock with the external environment. Whereas the peripheral clocks can be entrained by other signals such as diet, feeding regime, hormones, and microbiota-derived SCFAs apart from the regulation of the master clock [34,35]. The disorganization of the core clock altered the microbial composition, which may further result in intestinal dysbiosis and inflammatory diseases in mice [36]. The mammal circadian clock orchestrates the behavioral and physiological processes throughout the day. Essentially, both the epigenetics and transcriptome showed a significant rhythmic fluctuation. More specifically, about 45% of the gene expressed in a diurnal manner. These dynamic expressions of genes further determined the rhythmic variation of most physiological processes [37,38]. Interdependence cross talks between the gut microbiota and the host circadian clock play a critical role in maintaining the normal circadian rhythmicity of the host and the gut microbial community (Figure 2).

The host circadian clock was one of the most important factors that can regulate the microbial rhythmicity. It should be noted that the depletion of the circadian clock gene (*Bmal1* or *Per1/2*) has abrogated the rhythmic oscillations of the gut microbiota [8,16]. Moreover, the rhythmicity of microbial metabolites (e.g., SCFAs) was subsequently abolished in *Bmal1* gene depletion mice [13,14]. Consistently, chronically disrupted circadian rhythm induced by shifting the light: dark regime, jet lag, and shift work have influenced the oscillation pattern of gut microbiota [8]. However, the lost rhythmicity of gut microbiota and its metabolites is rescued by manipulating the feeding patterns. The findings suggest that the diurnal rhythmicity of gut microbiota can independently exist without the circadian clock [10,13]. The possible explanation was that a disrupted circadian clock affected the diurnal feeding rhythm in mice [39], which further changed the composition and diurnal rhythmicity of the gut microbiota [8,9,16].

In turn, the rhythmicity of the gut microbiota can influence the normal function of the host circadian clock. For example, the rhythmic adherence of microbiota affects the circadian transcriptome in the intestine. The fluctuations of the microbial metabolites can rhythmically program the liver transcriptome [10]. Furthermore, using a mono-colonized bacteria, Thaiss et al. (2016) indicated that the rhythmic bacterial adherence, not the presence of gut microbiota, programmed the rhythmic transcription in the peripheral circadian clock [10]. Notably, the expression of the core clock genes was disrupted in germ-free mice with decreased *Bmal1* and *Cry1* transcripts and increased *Per1* and *Per2* transcripts [40].

SCFAs might be one of the possible synchronizers of peripheral circadian clocks [11]. Both in-vivo and in-vitro studies indicated that the administration of SCFAs could indirectly shift the oscillation phase of the peripheral clock in the liver in a rhythmic manner [17]. Thus, SCFAs might be essential in the dynamic interactions between gut microbiota and the circadian clock in maintaining the homeostasis of normal physiology and synchronizing circadian activities. Through histone deacetylase, the gut microbiota can rhythmically regulate the host metabolism and affect the diurnal oscillations of the host physiology and the susceptibility to pathologies [41]. The direct evidence was that the gene expression profile in the liver of the germ-free mice was different from that in the normal mice despite the diet [11]. Intriguingly, microbial butyrate can regulate the rhythm patterns of *Per2* and *Bmal1* in the peripheral circadian clocks, implying that the microbiota indirectly modulates circadian rhythms via metabolites [11]. In addition, gut microbiota can regulate the intrinsic *Nfil3* circadian rhythms of the intestinal epithelial cell through type 3 innate lymphoid cells [42].

Polyamines are supposed to be primarily derived from diet and the gut microbiota, as the limitation of polyamines from the diet or microbiota has significantly decreased the circulating polyamine [43,44,45]. Compelling evidence suggests that polyamines, as pleiotropic signaling molecules, have been involved in various physiological and pathological processes [46,47]. More noteworthy, polyamines exhibit diurnal fluctuation. Changing the levels of polyamines could modulate the circadian period by regulating the interaction between the repressors of core clock PER2 and CRY1 both in in-vivo and in-vitro studies [23]. In addition to participating in carbohydrate metabolism and amino acid metabolism, the gut microbiota also plays an essential role in lipid metabolism, remarkably so, in pathways concerning the synthesis and oxidation of fatty acids [48,49]. The lipid concentrations in the liver and bloodstream were also oscillating rhythmically [50,51]. Choline trimethylamine-lyase derived from the gut microbiota is a rate-limiting enzyme transforming choline into trimethylamine. Surprisingly, inhibiting choline trimethylamine-lyase significantly reduced atherosclerosis and thrombosis and improved obesity in mice through remodeling the host peripheral circadian clocks [52,53,54].

Moreover, as emulsifiers in lipid metabolism, bile acids play critical roles in maintaining host metabolic homeostasis due to regulating the metabolism pathways related to cholesterol, triglyceride, and glucose metabolism [55,56]. Bile metabolism is in a time-of-day manner due to the need to coordinate metabolic responses to food intake, enabling the esterification and absorption of dietary fats and lipids [57,58]. The metabolism of bile acids, primarily secondary bile acids, is regulated by the gut microbiota [59,60]. Also, relevant findings suggested that the serum bile acids peaked at the beginning and end of the dark phase, while the serum secondary bile acids peaked at the beginning of the dark phase [61,62]. In addition, the microbial bile salt hydrolase significantly regulated the transcription of circadian rhythm genes such as *Dbp* and *Per1/2* in the liver or small intestine [63].

Despite that, the influencers of the microbial rhythmicity are primarily relatively well understood. However, the forming mechanism of the microbial rhythmicity remains unclear. It remains to clarify whether the gut microbial rhythmicity is self-sustained via the internal cellular clock of each microbe, whether the gut microbial rhythmicity is controlled by the peripheral circadian clock or the core circadian clock by affecting the host behavior such as activity and feeding rhythms? or whether the gut microbial rhythmicity is controlled by dynamic nutrition?

## 5. The Fundamental Implications of the Gut Microbiota Rhythmicity on Host Metabolism and Physiology

### 5.1. Disrupted Rhythmicity of Gut Microbiota in Obesity and Type-2 Diabetes Models

Obesity and type-2 diabetes, induced by aberrant glycolipid metabolism, are closely related to the gut microbiota. The higher prevalence is due to the high-fat and high-calorie diet in modern society. Growing evidence has linked the chronic metabolic disease of obesity and type-2 diabetes with gut microbiota [64,65,66,67,68]. Specifically, the gut microbiota plays a crucial role in modulating the process of fat storage, increasing energy harvesting, and regulating the formation of certain substrates [69,70]. Notably, type-2 diabetes might disturb the composition of gut microbiota and its corresponding function [71].

Further, recent research has revealed that metabolic diseases including obesity and type-2 diabetes might disrupt the diurnal rhythmicity of gut microbiota [12]. Especially, a high-fat diet has diminished the percentage of cyclical OTUs in mice and might change the fluctuation mode of the gut microbiota [15,72,73]. More specifically, the relative abundances of the dominant phyla Firmicutes and Bacteroidetes all underwent rhythmicity in the control group, whereas the rhythmic fluctuation of Firmicutes was lost in the high-fat diet group. However, the loss was rescued by melatonin, which is secreted by the pineal gland and plays a vital role in the circadian clock system [73,74,75]. Interestingly, the secretion of melatonin was also correlated with the risk of developing type 2 diabetes [76] and might prevent the incidence of obesity [77]. Beli et al. (2019) found that the diurnal rhythmicity of gut microbiota was disrupted in type-2 diabetes mice and the arrhythmia was correlated with the circulating metabolites in histidine, cysteine, and methionine-related metabolic pathways [78]. Further, Reitmeier et al. (2020) identified an arrhythmic gut microbiome in people with type-2 diabetes, mainly *Bifidobacterium longum*, *Clostridium celatum*, *Intestinibacter bartlettii*, *Romboutsia ilealis*, *Fecalibacterium prausnitzii*, and *Escherichia coli* based on a cohort distributed around Augsburg in Germany [12]. Nowadays, jet lag and shift work are normal in modern fast-paced lifestyles. However, it has been linked to the disrupted biological rhythm of both the host and the gut microbiota, which may further increase the risk of obesity and type 2 diabetes and imply the possible interactions between the host circadian rhythms and the gut microbiota rhythmicity [8].

### 5.2. The Role of Gut Microbiota Rhythmicity in the Regulation of Host Physiology, Immune and Metabolism

It is worthy to note that the gut microbiota rhythmicity can influence the host physiology and metabolism. Thaiss et al. (2016) revealed that microbiota diurnal rhythmicity programmed host transcriptome oscillations [10]. The liver is the most important metabolic organ in the body. However, the antibiotic intervention has reprogrammed the liver’s transcriptome oscillations, implying the systemic role of gut microbiota and its rhythmicity [10]. Blood pressure, as one of the most important physiological parameters for the body, has immense importance for predicting cardiac health and vascular status. Interestingly, blood pressure also showed a diurnal oscillation with a nadir at night and a peak during the day [79]. Abnormal blood pressure rhythm may increase the risk for cardiovascular diseases [80]. Chakraborty et al. (2020) found that the diurnal rhythm of blood pressure was synchronously related to the diurnal microbial shift [81]. In addition, plasma SCFAs exhibited diurnal oscillation in individuals who worked during the day, whereas SCFAs lost the 24 h rhythmicity in shift work subjects [82]. Unexpectedly, researchers further found that the concentrations of plasma SCFAs were negatively correlated with colonic permeability, which has fundamental implications for maintaining intestinal homeostasis [83,84]. Acetaminophen is ubiquitously used as an antipyretic and analgesic drug, whereas an overdose of acetaminophen would lead to severe hepatotoxicity [85,86]. Accumulating evidence has revealed that the hepatotoxicity induced by an overdose of acetaminophen exhibited diurnal differences: taking acetaminophen at night indued more serious liver injury [87,88]. Unexpectedly, diurnal variation of the hepatotoxicity abrogated in germ-free and antibiotics-treated mice suggests the important role of microbiota in drug metabolism [11]. Further research found that the diurnal variation of microbial metabolite 1-phenyl-1,2-propanedione mediated the diurnal variation of liver damage induced by overdosing of acetaminophen [89]. Besides, the microbiota rhythmicity also affected the susceptibility of a host to infection by pathogens. Bellet et al. (2013) found that the invasive capacity of *Salmonella typhimurium* has significant diurnal variation [90]. Meanwhile, antimicrobial peptide, which was supposed to be resistant to pathogen invasion and has fundamental implications for innate immunity, showed a rhythmic pattern [91]. So far, there is no evidence of the relationship between microbial rhythmicity with vaccine efficacy. However, convincing evidence has shown that microbiota composition can modulate the vaccine efficacy, and the antibody responses also underwent diurnal oscillation [92,93]. Importantly, metabolic pathways such as lipid metabolism and amino acid metabolism, which were closely related to certain gut microbiota and corresponding metabolites, also significantly oscillated in the day [10,50,51].

## 6. Developing Chronotherapy

With the continuous progress of chronobiology, therapies applying chronobiology have gradually emerged and developed into chronotherapy. The term chronotherapy refers to orchestrating therapeutics such as medications with the chronobiologic rhythms of the body to optimize ideal efficacy or reduce complications [94]. To date, the potential interventions for chronotherapy are as follows: (1) therapies concerning time administration of taking medication, eating or being fed, and lighting; (2) candidate hormones; (3) candidate nutrients. Considering the body complexity and the effectiveness of these interventions, it is necessary to develop comprehensive treatment packages including two or more interventions.

### 6.1. Time-Administration Therapy

Increasing evidence suggests that certain diseases such as myocardial infarction, acute cardiovascular diseases, and stroke occur predominantly during the light phase [95,96]. More specifically, a higher pro-inflammatory response was induced by the invasion of *Salmonella typhimurium* during the early rest period in the mice model compared with the other times of the day [90]. In addition, the side effects of some drugs (e.g., the hepatotoxicity induced by an overdose of acetaminophen) also exhibited robust rhythmicity [89]. Therefore, time is an important factor in maintaining normal physiology, preventing certain diseases, and avoiding drug toxicity. As the most important environmental cue, light is a primary zeitgeber that entrains/resets the circadian system both for the host and the gut microbiota [28,97]. Unfortunately, light pollution and shift work significantly disrupted the normal biological rhythmicity of the host and the microbiota. These arhythmicities may induce serious diseases including cancer, cardiovascular disease, depression, obesity, and diabetes [98,99]. Targeting these problems, a reasonable light exposure regime might be the best solution. However, in the fast-paced modern society, such activities are inevitable for humans. Fortunately, as a determinant of microbial rhythmicity, time-restricted feeding can entrain the peripheral clock [100,101,102]. Thus, imposed feeding-fasting rhythm can improve the metabolic disorders induced by shift work. In addition, auxiliary hormone and nutritional intervention may improve the metabolic disorder induced by the dysregulation in the diurnal rhythms, which we will further discuss in the following sections.

### 6.2. Candidate Hormone for Chronotherapy

Secreted by the pineal gland in the brain, melatonin is a neuro-hormone with pleiotropic physiological roles in maintaining host circadian and seasonal rhythms, modulating sleep and wakefulness cycle, regulating neuroendocrine actions, improving immune status, and eliminating free radicals [103,104]. As a key regulator of the circadian clock, melatonin exhibited robust diurnal rhythmicity. Stimulated by darkness, melatonin secretion increased in the darkness and peaked in the middle of the night, then decreased during the daytime to orchestrate the sleep–wake circle [105]. Light stimulus enhances the breakdown of melanopsin in retinal photoreceptive ganglion cells and decreases the synthesis of melatonin [106]. It should be noted that the administration of melatonin has rescued the dysbiosis in gut microbial rhythmicity caused by a high-fat diet [73]. Further, melatonin was revealed to synchronize the normal blood pressure rhythm in patients suffering from essential hypertension [107]. However, the dosage of melatonin and administration time should be strictly controlled [108].

### 6.3. Candidate Nutrients for Chronotherapy

Increasing evidence has indicated that diet, more precisely nutrients, was the most critical determinant that shapes the gut microbiota configuration [109]. Accordingly, studies have demonstrated that nutrients play vital roles in maintaining the normal rhythmicity of gut microbiota. Unexpectedly, convincing evidence suggested that a high-fat and sucrose diet attenuated the microbiota rhythmicity [9,25]. Fortunately, these adverse impacts on the rhythmic oscillation of gut microbiota and the circadian clock can be improved by certain functional nutrients or food additives. More specifically, the administration of probiotic *Lactobacillus reuteri* could improve the adverse effects of the high-fat diet [72]. Besides, as a natural antioxidant, oolong tea polyphenols can also enhance the disrupted diurnal rhythmicity of specific intestinal microbiota and alleviate the disordered rhythm of the expression of hepatic clock genes induced by constant dark in mice [27]. In addition, methionine exerts vital functions in regulating physiological processes concerning lipid metabolism, innate immune responses, and nutrient digestion, activating endogenous antioxidant enzymes and decreasing DNA damage and carcinogenic processes [110,111]. Wang et al. (2020) found that a methionine-restricted diet has partially restored the arhythmicity of the gut microbiota induced by a high-fat diet in mice [15].

## 7. Conclusions and Perspectives

Processes in physiology, pathology, metabolism, and immunology in mammals exhibited diurnal fluctuation [89,90,95,96]. These oscillations are under strict control of the circadian clock system. Importantly, well-established evidence suggests that the gut microbiota also underwent significant fluctuation at abundance, functional, and compartmental levels. Moreover, there is potential crosstalk between the gut microbiota rhythmicity and the host circadian rhythms in maintaining host health and metabolism. However, the formation mechanism of gut microbial rhythmicity is yet not precise. How the gut microbial rhythmicity contributes to beneficial effects in the host remains unclear. The regulatory role of these influencers on the dynamics of specific microbial taxa needs further investigation. Thus, future research should focus on the fundamental aspects of gut microbial rhythmicity, such as the formation mechanisms, the implications for the host, and their interactions with the host circadian clock. Insights into these questions facilitate a deeper understanding of the gut microbiota community and the development of chronotherapy targeting the gut microbiota.

## Data Availability

Not applicable.
